# Age-adjusted 5-factor modified frailty index as a valuable tool for patient selection in bilateral simultaneous total knee arthroplasty

**DOI:** 10.1038/s41598-024-65719-5

**Published:** 2024-06-25

**Authors:** Yuichi Yamaguchi, Yosuke Matsumura, Masanori Fujii, Shuya Ide, Tatsuya Sakai, Satomi Nagamine, Shuichi Eto, Takafumi Shimazaki, Tomonori Tajima, Masaaki Mawatari

**Affiliations:** 1 Department of Orthopaedic Surgery, Faculty of Medicine, Saga University, 5-1-1 Nabeshima, Saga, 849-8501 Japan; 2 Tsuruta Orthopaedic Clinic, Saga, Japan; 3 Department of Orthopaedic Surgery, National Hospital Organization Saga National Hospital, Saga, Japan; 4 Department of Orthopaedic Surgery, Taku City Hospital, Saga, Japan; 5 Department of Orthopaedic Surgery, JCHO Saga Central Hospital, Saga, Japan

**Keywords:** Total knee arthroplasty, Bilateral simultaneous, Complications, Frailty index, Risk factor analysis, Osteoarthritis, Musculoskeletal abnormalities, Risk factors

## Abstract

Although bilateral simultaneous total knee arthroplasty (BSTKA) is an effective treatment for bilateral knee osteoarthritis, safety concerns and lack of precise patient selection criteria persist. The purpose of this retrospective study was to determine the complication rate and the role of frailty in patient selection for BSTKA. We analyzed data from 434 patients who underwent BSTKA between February 2012 and January 2021, examining demographic factors and preoperative blood test results. Complications occurred in 77 patients (18%), with anemia requiring transfusion being the most common (26 patients, 5.9%). In the univariate analysis, age ≥ 75 years, age-adjusted Charlson Comorbidity Index ≥ 5, age-adjusted 5-factor modified Frailty Index (aamFI-5) ≥ 3, hemoglobin ≤ 11.0 g/dL, albumin ≤ 3.5 g/dL, estimated glomerular filtration rate < 45 ml/dl/1.73 m^2^, and D-dimer ≥ 2.0 μg/mL contributed to postoperative complications (p < 0.05). Multivariate analysis identified aamFI-5 ≥ 3 as an independent risk factor (p = 0.002). Our findings underscore the practical utility of aamFI-5 in predicting complications after BSTKA, providing valuable guidance to surgeons in the selection of BSTKA candidates and ultimately improving clinical outcomes.

## Introduction

Bilateral simultaneous total knee arthroplasty (BSTKA) has emerged as an effective procedure for patients with bilateral knee osteoarthritis, offering advantages such as shorter hospital stay and faster postoperative functional recovery compared to staged bilateral TKA^1,2^. It has also been shown to be a cost-effective procedure that benefits both patients and healthcare providers, with the potential to reduce healthcare costs^3^. However, the safety profile of BSTKA remains controversial, given its documented increased risk of postoperative complications, including venous thromboembolism, allogenic blood transfusion, cardiorespiratory and neurologic complications, and prosthetic infection, compared with unilateral or staged bilateral TKA^4–9^.

Preoperative patient characteristics, including age, general condition, comorbidities, and malnutrition, have been identified as risk factors for postoperative complications after TKA^7,10–12^. In this context, various scoring systems have been utilized to assess the risk of complications after TKA, including the Charlson Comorbidity Index (CCI), Age-adjusted CCI (ACCI), and American Society of Anesthesiologists (ASA) score^10,12–15^. However, the lack of validated risk stratification models specifically tailored to facilitate patient selection for BSTKA remains a notable gap in current orthopaedic practice.

Recent studies have convincingly demonstrated the pivotal role of frailty as a significant contributor to postoperative complications in various orthopaedic conditions^16–20^. Although several methods exist to quantify frailty^21^, the 5-item modified Frailty Index (mFI-5) has gained prominence in recent years due to its accepted validity and simplicity^16–20^. In particular, mFI-5 has the inherent advantage of extracting all its variables from patient interviews. Its ability to stratify risk and identify patients at high risk for adverse events is invaluable, not only to the patient and surgeon, but also to healthcare systems.

Most recently, the age-adjusted mFI-5 (aamFI-5), which incorporates chronological age into the previously described mFI-5, has demonstrated potential as a robust risk stratification tool for patients undergoing primary hip and knee arthroplasty^22,23^. However, its utility and specific discriminatory ability in predicting postoperative complications in a subset of BSTKA remains unexplored. This study aimed to determine (1) the incidence of postoperative complications after BSTKA and (2) the contributors to postoperative complications after BSTKA using a comprehensive risk factor analysis including aamFI-5. We hypothesized that aamFI-5 would outperform alternative scoring systems, such as ACCI or ASA score, in predicting postoperative complications in patients undergoing BSTKA.

## Patients and methods

This study was approved by the institutional ethical review board of the Faculty of Medicine, Saga University (No. 2022-07-R-04), and the requirement for informed consent of the participants was waived by the institutional ethical review board of the Faculty of Medicine, Saga University, due to the retrospective nature of the study.

### Patients

We performed a retrospective analysis of clinical data on BSTKA using a prospectively maintained arthroplasty database at our institution. Of 586 patients (1172 knees) who underwent bilateral TKA at our institution between February 2012 and January 2021, patients who underwent BSTKA were included in this study. Patients without at least one year of follow-up were excluded from the study. There were no clear selection criteria for BSTKA during the study period. Patients with symptomatic bilateral end-stage knee osteoarthritis were offered the option of undergoing either BSTKA or staged bilateral TKA, and BSTKA was selected primarily on the basis of patient preference.

### Data collection

We performed a detailed review of the medical records to collect patient demographics, preoperative blood test results, and any non-mechanical complications that occurred within one year of the BSTKA procedure. Preoperative demographic parameters included age, sex, body mass index (BMI), diagnosis, ASA score, ACCI, and aamFI-5. The mFI-5 is a composite score derived from comorbid diagnoses, including (1) diabetes mellitus, (2) hypertension requiring treatment, (3) chronic obstructive pulmonary disease, (4) congestive heart failure, and (5) non-independent functional status. Each comorbid diagnosis contributes one point to the total score. To account for the influence of older age on frailty, the aamFI-5 incorporates age by adding one additional point to the mFI-5 (for a total possible score of 6) for patients aged ≥ 73 years^23^. To establish cut-off values for numerical parameters indicating complication risk, we followed previous studies and defined them as follows: age ≥ 75 years^10,24^, BMI ≤ 18.5 kg/m^2^ or BMI > 25 kg/m^2^^25^, ASA score ≥ 3^10^, ACCI ≥ 5^12^, and aamFI-5 ≥ 3^22,23^. In addition, we evaluated the following preoperative blood test results as dichotomous variables using predetermined cut-off values for risk stratification: white blood cells (WBC) ≤ 3000 /mm or WBC ≥ 10,000 /mm, hemoglobin ≤ 11.0 g/dL, platelets < 100,000 /μL, C-reactive protein (CRP) ≥ 1.0 mg/dl, total protein (TP) < 6.5 g/dL, albumin ≤ 3.5 g/dL, estimated glomerular filtration rate (eGFR) < 45 ml/dl/1.73 m^2^, and D-dimer ≥ 2.0 μg/mL. Surgical parameters included operative time and perioperative visible blood loss. Visible blood loss included both intraoperative bleeding, represented by blood volume in surgical sponges and suction, and postoperative bleeding, quantified by volume in surgical drains. Postoperative surgery-related complications within the first year after BSTKA were categorized as follows: delayed wound healing, prosthetic infection, anemia requiring transfusion, symptomatic thrombosis (deep vein thrombosis and pulmonary embolism), cardiovascular complication, renal complication, respiratory complication, gastrointestinal disorder, and delirium. Transfusion decisions were based primarily on clinical signs of anemia, hemodynamic instability, or a postoperative hemoglobin < 8.0 g/dL.

#### Surgical technique

All BSTKA procedures were performed by a team of two experienced surgeons using a medial parapatellar approach. During the BSTKA procedure, tourniquets were applied to both legs to maintain a bloodless field. Specifically, the tourniquet on the first leg was inflated just prior to skin incision. After femoral resection of the first leg was completed, the tourniquet on the second knee was inflated, and the procedure on the second knee was initiated. After wound closure of each leg, the corresponding tourniquet was deflated. This protocol was maintained to minimize the total tourniquet time while ensuring the efficiency and safety of the procedure; the tourniquet time per knee was approximately 70 min. The distal femur and proximal tibia were cut perpendicular to their respective mechanical axes, with a 5-degree posterior slope in the tibia. The posterior femoral condyles were cut parallel to the surgical epicondylar axis. Patellar replacement was based on the severity of cartilage degeneration. An intra-articular suction drain was placed and removed on postoperative day 1. No cell saver device was used in these procedures.

Consistent postoperative management protocols were followed for all patients. Patients were allowed to begin gradual range of motion and full weight-bearing gait on the day after surgery. All patients received intravenous prophylactic antibiotics until postoperative day 1. Oral edoxaban tosilate hydrate was administered on postoperative days 1–14.

### Statistical analysis

Using the data described above, we performed (1) descriptive statistics on the occurrence of complications within the first year after BSTKA and (2) risk factor analysis, including patient demographic parameters and preoperative blood test data, to determine their association with the occurrence of complications.

Numerical parameters were presented as either mean (standard deviation) or median (range), depending on their distribution and homoscedasticity (Shapiro–Wilk test and f-test). The univariate logistic regression model was used to screen for factors associated with postoperative complications among the demographic parameters and preoperative blood tests. The significant factors were then examined using a multivariate logistic regression model to determine their independent influence on postoperative complications. The significance level was set at p < 0.05 for all tests. Statistical analyses were performed using JMP® Version 17.0 (SAS Institute Inc., Cary, NC, USA).

### Ethical approval

Each author certifies that his/her institution approved the human subject protocol for this investigation and that all investigations were conducted in conformity with the 1964 Helsinki Declaration. Ethical approval for this study was obtained from the institutional ethical review board of the Faculty of Medicine, Saga University (No. 2022-07-R-04), and the requirement for informed consent of the participants was waived by the institutional ethical review board of the Faculty of Medicine, Saga University, due to the retrospective nature of the study.

### Consent to participate and publish

Ethical approval for this study was obtained from the Faculty of Medicine, Saga University (No. 2022–07-R-04).

## Results

A total of 472 patients (944 knees, 81%) underwent BSTKA during the study period and were initially reviewed. Based on the exclusion criteria, 38 patients (76 knees) without at least one year of follow-up were excluded, leaving 434 patients (868 knees, 92%) eligible for this study (Table 1). The demographics of these patients were predominantly female (357 patients, 82%), with a median age of 76 years (IQR, 70–80 years). The median follow-up term was 47 months, ranging from 12 to 110 months. Posterior stabilized implants were used in all cases: Persona, LPS-Flex (ZimmerBiomet, Warsaw, US) in 177 patients (41%), Triathlon (Stryker, US) in 127 (29%), BS5 (Kyocera, Kyoto, JPN) in 81 (19%), Tri-MAX (MDM, Tokyo, JPN) in 19 (4.4%), NexGen (ZimmerBiomet, Warsaw, US) in 18 (4.1%), Vanguard (ZimmerBiomet, Warsaw, US) in 7 (1.6%), Initia (Kyocera, Kyoto, JPN) in 5 (1.1%). The median operative time was 109 min (IQR, 99–120 min) and the median perioperative visible blood loss was 690 ml (IQR, 438–910 ml). Postoperative surgery-related complications occurred in 77 patients (18%) within one year after BSTKA: all complications occurred within 90 days of surgery, with the exception of two cases where prosthesis infection occurred 4 months after surgery. The most common complication was anemia requiring blood transfusion, which occurred in 26 patients (5.9%), followed by delirium in 12 patients (2.8%), and delayed wound healing, and cardiovascular complication in 11 patients each (2.5%) (Table 2). There were no deaths within one year after BSTKA, while 7 patients (1.6%) died during the observation period unrelated to surgery.

**Table 1:** Patient demographic and surgical parameters (n = 434 patients [868 knees]).

Parameter	Median (interquartile range)/numbers (%)
Age* (years)	76 (70–80)
Sex^†^	
Male	77 (18)
Female	357 (82)
Body mass index* (kg/m^2^)	26 (24–29)
Diagnosis^†^	
Osteoarthritis	416 (96)
Rheumatoid arthritis	18 (4.1)
American society of anesthesiologists score^†^	
1	67 (15)
2	341 (79)
3	26 (6)
Age-adjusted Charlson comorbidity index^†^	
0–2	52 (12)
3–4	267 (62)
≤ 5	115 (26)
Age-adjusted 5-item modified Frailty Index^†^	
0	46 (11)
1	141 (32)
2	189 (44)
3	52 (12)
4	6 (1.4)
Follow-up period* (months)	47 (24–71)
Operation time* (min)	109 (99–120)
Perioperative visible blood loss* (ml)	690 (438–910)
Intraoperative bleeding* (ml)	0 (0–0)
Postoperative bleeding* (ml)	680 (430–910)

*Values are presented as the median (interquartile range).

^†^Values are presented as numbers (%).

**Table 2:** Postoperative complications (n = 434 patients [868 knees]).

Complications	Number of patients (%)
Anemia requiring transfusion	26 (5.9)
Delirium	12 (2.8)
Delayed wound healing	11 (2.5)
Cardiovascular complication	11 (2.5)
Congestive heart failure	9 (2.1)
Arrhythmia	2 (0.5)
Symptomatic thrombosis	8 (1.8)
Pulmonary embolism	6 (1.4)
Deep vein thrombosis	2 (0.5)
Prosthesis infection	7 (1.6)
Gastrointestinal disorder	6 (1.4)
Gastrointestinal ulcer	3 (0.7)
Cholecystitis	3 (0.7)
Renal complication	4 (0.9)
Acute kidney failure	4 (0.9)
Respiratory complication	3 (0.7)
Pneumonia	3 (0.7)

Univariate logistic regression analysis revealed that age ≥ 75 years (p = 0.004), ACCI ≥ 5 (p = 0.003), aamFI-5 ≥ 3 (p < 0.001), hemoglobin ≤ 11.0 g/dL (p < 0.001), albumin ≤ 3.5 g/dL (p = 0.018), eGFR < 45 ml/dl/1.73 m^2^ (p = 0.003), and D-dimer ≥ 2.0 μg/mL (p = 0.001) were associated with postoperative complications after BSTKA. Upon analyzing these seven factors together, only aamFI-5 ≥ 3 (p = 0.002) emerged as an independent risk factor for complications, with an odds ratio of 3.0 (Table 3). The complication rate in patients with aamFI-5 ≥ 3 (23 of 58 patients, 40%) was higher than that in patients with aamFI-5 < 3 (54 of 376 patients, 14%) (p < 0.001).

**Table 3:** Logistic regression analysis of risk factors for complication after bilateral simultaneous total knee arthroplasty (R^2^ = 0.09, p < 0.001).

Parameter	Univariate		Multivariate	
	Odds ratio (95% CI)	p-value	Risk ratio (95% CI)	p-value
Demographic factors				
Age ≥ 75 (years)	2.2 (1.3–3.8)	0.004	1.4 (0.8–2.6)	0.257
Sex (men)	1.3 (0.7–2.4)	0.442		
Body mass index ≤ 18.5 or > 25 (kg/m^2^)	0.9 (0.5–1.4)	0.575		
Diagnosis (rheumatoid arthritis)	2.4 (0.9–6.7)	0.085		
ASA score ≥ 3	1.4 (0.6–3.7)	0.464		
ACCI ≥ 5	2.2 (1.3–3.7)	0.003	1.0 (0.5–2.1)	0.967
aamFI-5 ≥ 3	3.9 (2.2–7.1)	< 0.001	3.0 (1.5–5.9)	0.002
Preoperative laboratory tests				
WBC ≤ 3000 or ≥ 10,000 (/mm^3^)	0.7 (0.1–5.4)	0.697		
Hemoglobin ≤ 11.0 (g/dL)	3.4 (1.6–7.0)	< 0.001	1.9 (0.8–4.5)	0.121
Platelet ≤ 100,000 (/μl)				
CRP ≥ 1.0 (mg/dl)	2.3 (0.9–5.8)	0.083		
Total protein < 6.5 (g/dL)	1.2 (0.4–3.6)	0.786		
Albumin ≤ 3.5 (g/dL)	2.7 (1.2–6.0)	0.018	1.2 (0.4–3.2)	0.741
eGFR < 45 (ml/dl/1.73m^2^)	2.8 (1.4–5.6)	0.003	1.8 (0.7–4.6)	0.203
D-dimer ≥ 2.0 (μg/mL)	2.9 (1.5–5.5)	0.001	2.1 (1.0–4.3)	0.053

*ASA* American Society of Anesthesiologists, *ACCI* age-adjusted Charlson Comorbidity Index, *aamFI-5* age-adjusted 5-factor modified frailty index, *WBC* white blood cells, *CRP* C-reactive protein, *eGFR* estimated glomerular filtration rate.

## Discussion

BSTKA represents a cost-effective option for patients with bilateral knee osteoarthritis, offering advantages such as shorter hospital stay and accelerated postoperative functional recovery compared to staged bilateral TKA^1–3^. However, the safety profile of BSTKA remains controversial, particularly in high-risk patients^4–9^, and the lack of validated risk stratification models specifically tailored to BSTKA candidate selection underscores a critical gap in modern orthopaedic practice. This study is the first attempt to evaluate the applicability of the aamFI-5 in identifying suitable candidates for BSTKA by comparing the ACCI and ASA scoring systems. Our findings support the use of the aamFI-5 as an effective risk stratification tool for predicting postoperative adverse events in this population.

Patients often present with bilateral knee osteoarthritis, with approximately 40% requiring contralateral TKA within eight years of primary surgery^26^. Despite the acknowledged benefits of BSTKA, the predominant body of research demonstrates an increase in complications compared to unilateral TKA, with older patient age and associated comorbidities identified as primary risk factors^4,7–10,12,15,27^. On the other hand, various studies have compared the outcomes of simultaneous and staged bilateral TKA; however, no consensus has emerged regarding the safety and efficacy of either approach^1,2,5,6,9,28^. In the current study, the incidence of postoperative complications following BSTKA was 18%, which is consistent with previous studies that have reported rates ranging from 12 to 35%^10,12,28,29^. During the study period, 81% of patients opted for BSTKA, reflecting their preference and the overall healthier status of the group, with 79% of patients being ASA 2 and 15% ASA 1, underscoring the lower risk profile of our study population. Anemia requiring blood transfusion was the most common postoperative complication in our cohort, affecting 5.9% of patients, which is consistent with previous reports of increased transfusion rates in BSTKA^9,28^. However, it is noteworthy that previous studies have documented higher transfusion rates in the setting of BSTKA, ranging from 35 to 59%^28,30^. The relatively lower incidence observed in our study could be attributed to the implementation of adjunctive modalities, such as tranexamic acid administration and drain clamping. Although our protocol included surgical drains, emerging evidence suggests that the omission of surgical drains in TKA may reduce postoperative blood loss and transfusion rates without increasing the risk of complications such as infection or hematoma^31^. Further studies are required to confirm these benefits in the BSTKA setting.

Regarding patient demographic factors, univariate analysis showed that age ≥ 75 years, ACCI ≥ 5, and aamFI ≥ 3 were associated with postoperative complications following BSTKA in this study. The pathophysiology of BSTKA differs from that of unilateral TKA due to its greater impact on the cardiopulmonary system^9,27,28^. Therefore, when evaluating candidates for BSTKA, orthopaedic surgeons have typically employed conservative risk stratification methods to mitigate patient risk, such as avoiding patients with cardiopulmonary disease or advanced age^4,7–10,12,15^. Koh et al.^10^ also showed that age ≥ 75 years combined with ASA score ≥ 3 and concomitant cardiovascular disease, increased the risk of postoperative complications. Amit et al.^12^ found that BSTKA was associated with a significantly increased risk of cumulative total complications compared to unilateral TKA, especially in patients with ACCI ≥ 5.

Notably, multivariate analysis identified aamFI-5 as an independent risk factor for postoperative complications after BSTKA, with an odds ratio of 3.0. Patients with aamFI-5 ≥ 3 had an increased complication rate of 40% compared to 14% in patients with aamFI-5 < 3. Frailty is an established risk for patients undergoing primary TKA, with the mFI-5 prevailing due to its validity and simplicity^16,17^, although it overlooks age as a contributing factor. Frailty adversely affects outcomes after TKA in all age groups, although older and frail patients are more vulnerable. The aamFI-5 captures this aspect and has been shown to be a simple yet robust tool for risk stratification in patients undergoing primary total hip and knee arthroplasty^22,23^. The results of this study underscore the superior discriminative ability of the aamFI-5 compared to alternative scoring systems such as the ACCI and ASA scores in prognosticating postoperative complications in a subset of BSTKA.

Regarding preoperative blood tests, univariate analysis identified hemoglobin < 11.0 g/dL, albumin < 3.5 g/dL, eGFR < 45 ml/dl/1.73 m^2^, and D-dimer > 2.0 μg/mL as risk factors for postoperative complications after TKA. Johnson et al.^11^ identified hypoalbuminemia (albumin < 3.5) as the most potent risk factor for postoperative complications after TKA, emphasizing the need for preoperative nutritional optimization. Schwartz et al.^16^ showed that the coexistence of hypoalbuminemia (albumin < 3.5 g/dL) and frailty (mFI-5 < 2) was synergistically associated with mortality and complications after primary TKA. On the other hand, DeMik et al.^32^ observed that patients with preoperative anemia required more blood transfusions after primary and revision TKA. While none of the blood test results examined in this study were independent risk factors for complications following BSTKA, the results of previous and current studies consistently underscore an association between malnutrition and anemia and postoperative complications after TKA. Further studies are warranted to determine whether the integration of blood test results with age and comorbidity assessment could improve the accuracy of stratifying potential BSTKA candidates.

### Limitations

Despite the contributions of this study, its limitations must be acknowledged. First, its retrospective design, based on our institution's database and unmatched study groups, is a primary limitation. Nevertheless, the standardized 14-day postoperative hospital stay for all BSTKA patients in our healthcare system facilitated the early detection of most complications occurring within this timeframe. Second, although the sample size was substantial, it may not be sufficient to provide a robust power analysis. However, it is imperative to note that this study was conducted at a single institution, which mitigates potential biases from variables such as differences in surgical techniques, surgeon expertise, and management protocols, which are often encountered in multicenter or registry-based investigations. Third, the predominantly female demographics (82%) and the low percentage of patients with aamFI-5 ≥ 3 (13%) may affect the generalizability of our results. Despite a substantial sample size, a larger study with a more diverse demographic would be ideal to confirm the efficacy of the aamFI-5 in predicting surgical outcomes. Lastly, the one-year follow-up for postoperative complications, while thorough, carries the risk of including complications not directly related to surgery. However, we carefully excluded all late-onset conditions unrelated to BSTKA to maintain the specificity of our assessment of surgical outcomes. We believe that our extended follow-up allowed us to capture significant late complications, such as prosthesis infection, that occurred after the typical 90-day period, providing a comprehensive view of surgical outcomes and patient safety.

## Conclusion

In conclusion, this study demonstrated that the aamFI-5 is an effective risk stratification tool for predicting postoperative complications after BSTKA, outperforming the ACCI and ASA scoring systems. Our findings provide valuable guidance to surgeons in the selection of BSTKA candidates, ultimately leading to improved clinical outcomes.

## Data Availability

The datasets generated during and/or analyzed during the current study are available from the corresponding author upon reasonable request.
